# A five-year study on the epidemiological approaches to cholera in Iran

**Published:** 2016

**Authors:** Moharam Mafi, Mohammad Mahdi Goya, Massoud Hajia

**Affiliations:** 1 Center for Disease Control, Ministry of Health and Medical Education, Tehran, Iran.; 2 Research Center of Health Reference Laboratories, Ministry of Health and Medical Education, Tehran, Iran.

**Keywords:** Epidemiology, Risk factors, *Vibrio cholerae*

## Abstract

**Background:**

Cholera is considered a key indicator of social development but still is reported in various cities of Iran. The present study aimed to analyze the available information regarding cholera outbreaks since 2010 in Iran.

**Methods:**

All cases reported to the Center for Disease Control and Prevention of Ministry of Health and Education who had been confirmed as cholera cases by the Health Reference Laboratory, were entered into this study since 2010. A specific spreadsheet was designed to ensure the safe keeping of the patient records.

**Results:**

A total of 1522 patients were clinically diagnosed as cholera with laboratory confirmation over the study period. Cholera was detected in 26 Provinces and 115 cities during this period. Mean age of the patients was 35.1±17, both the Inaba and Ogawa strains were isolated. The highest mortality and the morbidity rate was 1.98% in 2013. The most cholera prevalent provinces in order of frequency were Baluchistan, Alborz, Gilan, Golestan and Qom, as well as Tehran. Inaba serotype was the most common cause of mortality and morbidity in 2013.

**Conclusion:**

These findings indicate significant outbreaks of cholera in some of the provinces of Iran and warrant appropriate treatment and preventive measures.

*Vibrio cholerae* is a natural inhabitant of the aquatic environment and also is the cause of most prevalent water-related infections in many regions of the world. Cholera has remained a major public health problem and is endemic in many countries from South Asia and the Bay of Bengal (1) to various parts of Africa. It spreads usually through contaminated food and water, often after civil unrest or natural disasters*, *although outbreaks may be induced mostly from contaminated seafood in Southeast Asia (2). Based on the World Health Report, it is estimated that approximately 3–5 million cholera cases occur every year globally. However, only a small portion of these cases have been reported to WHO. It is also estimated that 4.2% of the total cholera cases and 100,000 to 120,000 deaths reported globally originate in Asian countries (3, 4). The highest number of cholera cases have been reported in India (52.9%), followed by Afghanistan (29%) and Indonesia (22%) (3, 5, 6). *Epidemiological aspects of V. cholera* has been described in several studies for its survival in aquatic environments and also for its potential role as a reservoir in subsequent disease outbreaks. Influential parameters of cholera outbreaks were mostly studied for role of living in rural area and age risk group. Reported infectious mostly occur from summer until early fall (7, 8).

WHO recommends safe water supply and adequate sanitation and hygiene (WASH) as the main steps to prevent cholera (3). Therefore, cholera is a key indicator of social development. Among our neighboring countries, Pakistan is specifically at higher risk for waterborne diseases because it has an agricultural economy with one of the most expansive water distribution systems in the world. The Centers for Disease Control and Prevention (CDC) of the United States defined outbreak and epidemics as a sudden increase in the number of cases of a disease above what is normally expected in the population area (9). WHO defined alert for each outbreak. The defined alert for cholera reports one suspected case of cholera whereas outbreak is a confirmed case, or a cluster of three or more suspected cases in a single locality (10).

Primary health care coverage reported increased rate of cholera within last decade and provided some of the sanitary services such as safe drinking water in our region. But we are still facing with some local epidemics that occasionally spread to the vast area and engage many parts of Iran. Periodic outbreaks of cholera have been reported from Sistan and Balouchestan and other provinces in the southeast of Iran. Most of these outbreaks are believed to be linked to the cross-border movement of populations (11).

The present study aimed to perform the descriptive analysis of the available information regarding cholera outbreaks since 2010. 

## Methods

Any patient with signs and symptoms of cholera was considered as an alert in this study, whereas outbreak was defined as a confirmed case or a cluster of three or more cases in a single locality. All reports to the Center for Disease Control and Prevention of Ministry of Health and education that were confirmed as a cholera cases were entered into this study from 2010 to 2014.

**Identification and confirmation procedure of** ***V. cholerae*** **:** The *V. cholerae* isolates were diagnosed form sporadic and epidemic cases during these years which occurred in any provinces at their local laboratories. Based on the routine procedure, these isolates were transferred to the Health Reference Laboratory for re-identification as referral Laboratory for final confirmation using standard biochemical and bacteriological tests and were examined for specific serogroups by O1 polyvalent and Ogawa/Inaba monospecific antisera (BD, Becton Dickinson Co.USA) (12, 13).

**Data collection analysis** **:** All epidemiological data of cholera in sporadic and epidemic cases were obtained from registered documents of Center for Disease Control and Prevention of Ministry of Health and education since 2010 based on CDC and WHO definition. Analyzed data were saved on a computer. A spreadsheet with specific codes was used to ensure safekeeping the patient records. Descriptive statistic was used for expression of data 

## Results

**Figure 1:**
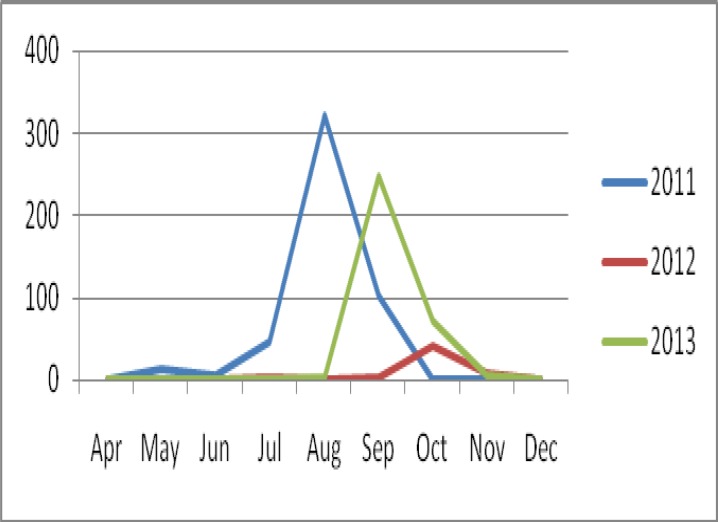
Seasonal distribution in those years with high infection rate (2011-2013)

A total of 1522 patients were clinically diagnosed as cholera with laboratory confirmation in the study period. The mean age of the confirmed patients was 35.1±17.11 but varied across different years. The peak of the infection varied from about August till November (figure 1 & table 1). 

The total number of infected women was slightly higher than men, although the rate of infection in males and females was different in the study period (table 2). Both Inaba and Ogawa strains were isolated from infected people in which 19.2% of specimens were Inaba and the rest were Ogawa. Frequency of the urban patients was obviously higher than those confirmed cases of rural areas (table 3). The highest mortality rate was 1.94% which was observed in 2013. All Inaba isolates were dominant in that year (table 4). The frequencies of cholera in Iranian patients and other nationalities are mentioned in table 5.

Cholera was observed in 26 Provinces and 115 cities during the study period. The high rate of infection was observed in Baluchestan/ Alborz/ Gila/ Gvlesta/ Qom as well as Tehran. Among those provinces located at the border of the country, Baluchestan was the only place with the highest rate of cholera (table 6). 

**Table 1:** Frequency the age of cholera patients from 2010 till 2014

**Age groups**	**2010**	**2011**	**2012**	**2013**	**2014**	**Total**
0-5	4	69	4	9	0	86
5-10	2	22	4	7	0	35
10-15	2	28	4	5	1	40
15-20	0	58	2	55	2	117
20-25	2	146	8	79	0	235
25-30	2	144	7	36	2	191
30-35	0	115	6	21	3	145
35-40	1	105	3	14	0	123
40-45	0	82	5	10	0	97
45-50	0	77	2	5	0	84
50-55	0	77	3	6	0	86
55-60	0	52	2	3	0	57
60-65	1	89	1	5	1	97
65-70	0	45	1	0	0	46
Over 70	1	79	1	2	0	83
Total	15	1188	53	257	9	1522
	21.2±16.96	37.65±17.57	29.5±16.51	25.52±11.7	28.66±13.74	35.1±17.11

**Table 2:** Frequency of male and female patients

	**2010**	**2011**	**2012**	**2013**	**2014**	**Total**
Men	10 (66.66%)	637 (53.64%)	39 (73.59%)	28 (10.9%)	5 (55.55%)	719 (47.27%)
Women	5 (33.33%)	551 (46.36%)	14 (26.41%)	229 (89.1%)	4 (44.45)	803 (52.73%)
Total	15	1188	53	257	9	1522

**Table 3:** Frequency of Inaba and Ogawa serotypes in the study period

	**2010**	**2011**	**2012**	**2013**	**2014**	**Total**
Inaba	0 (0.0%)	13 (1.1%)	20 (37.74%)	254 (98.83%)	5 (55.55%)	293 (19.2%)
Ogawa	15 (100%)	1175 (98.9%)	33 (62.24%)	3 (1.17%)	4 (44.45%)	1229 (80.8%)
Total	15 (100%)	1188 (100%)	53 (100%)	257 (100%)	9 (100%)	1522 (100%)

**Table 4:** Mortality rate from 2010 to 2014

	**2010**	**2011**	**2012**	**2013**	**2014**	**Total**
Cholera Infection	15	1188	53	257	9	1522
Mortality	0	5 (0.42%)	0	5 (1.94%)	0	11 (0.72%)

**Table 5:** Frequency of confirmed cholera cases in Iranian and other nationalities

	**2010**	**2011**	**2012**	**2013**	**2014**	**Total**
Iranian	11	1107	18	43	5	1183
Afghan	1	63	2	212	1	279
Pakistani	3	18	3	2	3	29
Iraq	0	0	30	0	0	30
Total	15	1188	53	257	9	1522

**Table 6:** Frequency and distribution of the cholera throughout the country

	**Provinces**	**2010**	**2011**	**2012**	**2013**	**2014**	**Total**
1	Baluchestan	10	35	20	142	3	210
2	South Khorasan	0		0	1	1	2
3	Khorasan Razavi	0	3	0	1	0	4
4	Hormozgan	0	4	0	8	0	12
5	Bushehr	1	1	1	0	3	6
6	Khuzestan	2	3	1	0	0	6
7	Lorestan	0	2	0	0	0	2
8	Kermanshah	0	0	2	0	0	2
9	Hamedan	0	1	0	0	0	1
10	Kordestan	0	0	23	0	0	23
11	West Azarbayejan	0	0	5	0	0	5
12	Ardebil	0	3	0	0	0	3
13	Zanjan	0	67	0	0	0	67
14	Qazvin	0	89	1	5	0	95
15	Markazi	0	11	0	0	0	11
16	Fars	0	3	0	8	0	11
17	Kerman	0	3	0	73	0	76
18	Esfehan	0	12	0	2	0	14
19	Yazd	2	4	0	0	0	6
20	Qom	0	138	0	3	0	141
21	Tehran	0	171	0	10	1	182
22	Alborz	0	233	0	2	1	236
23	Semnan	0	16	0	0	0	16
24	Mazandaran	0	3	0	0	0	3
25	Golestan	0	157	0	0	2	159
26	Gilan	0	229	0	0	0	229
	Total	15	1188	53	257	9	1522

## Discussion

Cholera is a preventable and treatable communicable infectious disease that still has remained a significant threat to public health particularly in developing countries of Asia and Africa. This disease is a key indicator of social development and the risk is highest in areas where basic infrastructure is not available. Endemic country of cholera is defined by World Health Organization as the one which has reported cholera cases in at least three of the five most recent years (14). Cholera has continuously been diagnosed in some parts of Iran in in spite of improving sanitary services and establishing accurate surveillance system in these recent years (15-17). It is believed that Iran is endemic country in the regions, although sometimes it is attributed to the cross-border movement of Afghan and Pakistan populations. The present study is an effort to analyze, the recent five years cholera outbreak throughout the country, since no systemic study has been conducted yet to identify the true distributive pattern of cholera in Iran in recent outbreaks.

Our results revealed serogroup O1 and biotype of cholera remained significant and predominant cause of cholera infection in Iran. Both Ogawa and Inaba were the main infectious serotypes. Ogawa serotype was the dominant type at the beginning of the study, while Inaba gradually specified higher position at the end of study. The highest mortality and morbidity rate of this study was observed, Inaba serotype was the dominant one (1.98%). This rate was remarkably lower than the neighboring countries (2, 18, 19).

The most common risk factors were contaminated water sources, heavy rainfall and flooding, and population dislocation. Various studies focused on these risk factors. It is believed that the epidemic regions are located near regional rivers and are characterized by sporadic outbreaks, which are likely to be initiated during episodes of prevailing warm air temperature with low river flows, creating favorable environmental conditions for growth of cholera bacteria. Availability of the risk factors accelerates interaction between contaminated water and human activities (20). Based on this consideration, Iran is not exempted to have a good suitable habitability for cholera although it is accepted as an endemic country at least in some regions. 

During the study period, twenty six provinces out of thirty one with only 115 cities faced with cholera in the study period. There is no report from the rest of the provinces (table 6). Documents showed the outbreaks started mainly from Baluchestan and the source of infection is in the neighboring countries which especially spread via foreign travelers to Iran. Paying attention to more control of the border is a strong influential factor to reduce the rate of infection particularly in spring and summer seasons. Improving the sanitary services in some parts of Baluchistan provinces is necessary to reduce the cholera rate as well.

The high rate of infection was observed in Baluchestan, Alborz, Golestan, Qom as well as Tehran. Among those provinces located at the border of the country, Baluchestan is the only place with the highest cholera rate. Qom and Alborz are the two next provinces with higher rate because of population of huge foreign travelers. Qom is known as the Islamic theological center for clergymen and Alborz has encouraging situation for temporary jobs. Golestan and Gilan are those provinces located in the northern part of Iran near Mazandaran Sea. The analyzed data revealed higher rate of infections compared with other provinces. This can be due to some risk factors such as heavy rainfall and huge tourists. It is believed that these two provinces have a good registration and reporting systems, so higher cholera reports is believed to carefully monitoring the disease in these regions. Analysis of the results shows the frequency of male and female patients are nearly similar at first glance. However, it is different in each year. Comparison of presented results (figure 1, table 1, and table 5) underlines the number of male patients and their mean age was remarkably higher in those years cholera started in the late summer. This reflecting strong role of the foreign travelers in outbreaks. 

**Conclusion: **Analyzed results suggest these that outbreaks have been linked to the cross-border movement of populations specifically in Baluchestan. The next target provinces were Qom and Alborz, causes necessity to watch over these provinces.

## Notes

**Funding:** The study was supported by the Iran Ministry of Health and Medical Education.

## Citation:

Mafi M, Goya M, Hajia M. A five-year study on the epidemiological approaches to cholera in Iran. Caspian J Intern Med 2016; 7(3): 162-167.
